# NupR Is Involved in the Control of PlcR: A Pleiotropic Regulator of Extracellular Virulence Factors

**DOI:** 10.3390/microorganisms13010212

**Published:** 2025-01-20

**Authors:** Jiaxin Qin, Ziqi Wang, Cheng Qian, Guohui Ji, Yizhuo Zhang, Zhanglei Cao, Bing Yan, Jun Cai

**Affiliations:** 1 Department of Microbiology, College of Life Sciences, Nankai University, Tianjin 300071, China; 2 Key Laboratory of Molecular Microbiology and Technology, Ministry of Education, Tianjin 300071, China; 3 Tianjin Key Laboratory of Microbial Functional Genomics, Tianjin 300071, China

**Keywords:** PlcR, NupR, virulence, glucose, *Bacillus thuringiensis*

## Abstract

NupR is a nucleoside permease regulator belonging to the GntR family, mainly regulating nucleoside transport in *Bacillus thuringiensis*. A conserved binding site for NupR was found in the promoter region of *plcR*. This study aimed to investigate the regulation of the virulence regulator PlcR by NupR and its impact on Bt virulence. We demonstrated that NupR can directly repress the expression of *plcR*. The expression of *plcR* can be induced by glucose and nucleosides. Glucose impacts the expression of *plcR* mainly through Spo0A, while the induction effect of nucleosides may be due to the production of ribose through nucleoside catabolism. In addition, NupR regulates the expression of the PlcR regulon, including hemolysin, phospholipase C, *papR*, and oligopeptide permease, which could result in the culture supernatant of BMB171 being less virulent to sf9 cells compared to the *nupR* knockout strain. The results combine the nutritional status of cells with virulence to form a regulatory loop, providing new ideas and research foundations for the study of bacterial virulence.

## 1. Introduction

The transcription factor PlcR regulates the expression of approximately 45 genes in the *Bacillus cereus* group of spore-forming Gram-positive bacteria [1,2]. In addition to the pathogenic *Bacillus cereus*, which causes foodborne and opportunistic infections, the *Bacillus cereus* group also includes six other species, such as the anthrax pathogen *Bacillus anthracis* (Ba) and the insect pathogen *Bacillus thuringiensis* (Bt). PlcR is truncated in Ba, rendering it inactive [1,3,4]. Most of the genes controlled by PlcR in *B. cereus* and *B. thuringiensis* encode proteins related to food supply and virulence (phospholipases, proteases, hemolysins, toxins, etc.), cell protection, and environmental sensing. Deletion of *plcR* significantly reduces the virulence of *B. thuringiensis* (against insects) and *B. cereus* (in mouse infection models) [5].

It has been demonstrated that PlcR can bind to DNA at specific sequences known as “PlcR boxes”, located upstream of the controlled genes and at varying distances in front of the −35 box of the promoter [1,6,7]. Transcription of *plcR* begins shortly before the stationary phase at T0 and reaches a plateau two hours later (T2) [8]. Transcription of *plcR* is autoinduced [8], and it is inhibited by the sporulation factor Spo0A [9]. In addition, the expression of *plcR* is also regulated by the YvfTU two-component signal system located near its genetic locus in *Bacillus cereus*. During the high expression of the PlcR regulon phase, the expression of *plcR* in the *yvfTU* mutant is only 50% of that in the wild-type strain. Moreover, the *yvfTU* mutant exhibits slightly lower virulence in the *Galleria mellonella* insect model than the wild strain [10]. It has been reported that the expression of *plcR* and PlcR-dependent genes in *B. cereus* requires the global regulator CodY [11,12]. The impact of CodY on the expression of virulence factors is not achieved through the direct binding of CodY to the promoter regions of *plcR* or PlcR-dependent genes. Instead, it participates in the expression of virulence factors in *B. thuringiensis* through its role in the import of the quorum-sensing signal peptide PapR.

PlcR requires activation by PapR. This peptide, which is expressed as a precursor under the control of PlcR, is exported outside the cell. After being processed by proteases such as NprB in the periplasmic space, PapR can become the mature pentapeptide PapR_7_, which accumulates gradually in the periplasmic space as a signaling molecule, responding to the density and state of the bacterial community and is imported into the bacterial cell through the bacterial surface oligopeptide permease OppABCDF after accumulating to a certain concentration. Once transported into the cell, PapR_7_ binds to PlcR, causing a conformational change in PlcR, forming a PlcR-PapR complex dimer that recognizes and binds to the target and activates the expression of downstream genes [6,13,14,15]. Thus, the three partners—PlcR, OppABCDF, and PapR—act as a quorum-sensing system.

Previous research has shown that in the *Bacillus thuringiensis* BMB171 strain, the GntR/HutC family transcriptional regulator NupR (nucleoside permease regulator) can directly bind to the 5’ noncoding region of *plcR*. The NupR transcriptional regulator is highly conserved in the *Bacillus cereus* group, and NupR-like proteins are also widely present in *Bacillus subtilis*, *Clostridium difficile*, *Pseudomonas aeruginosa*, and *Streptococcus pneumoniae*. Whether this protein is involved in regulating *plcR*, the mechanism of regulation, and its significance are still unknown [16].

Therefore, in this study, we investigated the regulatory effects of NupR on *plcR* and its regulon at different time points and assessed the impact of *nupR* deletion on the virulence of *Bt*. NupR (nucleoside permease regulator) can directly regulate the expression of four nucleoside permeases. Moreover, glucose can induce *nupR* expression through CcpA. Thus, we conducted further investigations into the response of *plcR* to glucose and nucleosides.

## 2. Materials and Methods

### 2.1. Bacterial Strains and Culture Conditions

The bacterial strains used in this research were stored in our laboratory at −80 °C and are listed in Supplementary Table S1. The strains were removed from the −80 °C freezer, activated in 5 mL Luria–Bertani (LB) liquid medium, and subsequently streaked onto LB agar plates containing the appropriate antibiotics. They were then cultivated in 5 mL LB medium containing the appropriate antibiotics for experimental investigations. *B. thuringensis* BMB171 and its derived strains were cultured in LB or Schaeffer’s sporulation medium (SSM) [17] at 28 °C with shaking at 200 rpm. *Escherichia coli* strains were cultured in LB media at 37 °C with shaking at 200 rpm.

### 2.2. RNA Extraction and RT–qPCR

The BMB171 strain and its derivative strains were cultured in SSM to the early stationary phase. Following the supplementation of the culture medium with the inducers, a 30 min induction period was implemented to allow for the activation of the targeted genetic pathways. The bacterial solution was centrifuged to remove the supernatant, and the cell pellet was resuspended in 1 mL of RNAiso Plus. Zirconium beads were added for cell disruption. After grinding, 200 µL of RNA extraction solution was added to the homogenate and it was mixed gently and allowed to stand for phase separation. It was centrifuged at 12,000 rpm for 20 min at 4 °C, and the upper aqueous phase was transferred to a new RNase-free 1.5 mL centrifuge tube. An equal volume of pre-chilled isopropanol was added and incubated at −20 °C to enhance RNA precipitation. It was centrifuged again at 12000 rpm for 20 min at 4 °C, the supernatant was discarded, and the pellet was washed twice with anhydrous ethanol. Finally, the centrifuge tube was placed in a 37 °C metal bath to evaporate any remaining alcohol. The RNA pellet was dissolved in RNase-free water and its concentration was determined.

For reverse transcription, 1 µg of RNA was converted to cDNA using a reverse transcription kit (Takara, Biotechnology Corporation, Dalian, China). The cDNA was then used as a template for quantitative PCR (qPCR) with TB Green Premix Ex Taq™ II (Tli RNaseH Plus) (Takara). The 16S rRNA gene was used as an internal control [16,18,19].

### 2.3. Determination of β-Galactosidase Activity

Overnight cultures were transferred to 100 mL of SSM or LB medium and cultured at 200 rpm and 28 °C until the cells reached the end of the exponential growth period (T0). A total of 1 mL of sample was taken to determine the OD_600_ of the bacterial solution. Then, 2 mL samples were taken and centrifuged to determine the β-galactosidase activity at the desired time. The method has been described elsewhere [16,20]. The cell pellet was resuspended in 1 mL of Z buffer; then zirconium beads were added and the cells disrupted using a tissue breaker. The resulting supernatant was collected as the reaction solution. Next, 800 µL of Z buffer was added to a 2 mL tube, which was placed in a 37 °C metal bath. Then, 200 µL of the reaction solution and 200 µL of ONPG was added to initiate the reaction, recording the start time as T0. Finally, 500 µL Na_2_CO_3_ (1M) was added to terminate the reaction, recording the stop time as T1. After the reaction was stopped, the optical density at 420 nm was measured. The Miller unit was calculated using the following formula: Miller unit = 1000 × OD_420_/(T1 − T0) × OD_600_ × V. V = 200 µL; T1 − T0 = reaction time.

### 2.4. Electrophoretic Mobility Shift Assay (EMSA)

The purification of NupR and the electrophoretic mobility shift assay were performed as previously described [16]. The purified NupR-His protein was incubated with FAM-labeled P*mogR* fragments in a buffer (10 mM Tris-HCl, pH 7.5, 50 mM NaCl, 0.5 mM dithiothreitol (DTT), and 4% glycerol) at 28 °C for 25 min. To verify whether the binding of the protein to DNA is specific, 3 µg of salmon sperm DNA and 3 µg of unlabeled P*mogR* DNA fragments were added separately. Next, the mixture was loaded onto a 6% native polyacrylamide gel, which had been pre-electrophoresed in 0.5× TBE buffer for 30 min. The gel was run at 140 V for 90 min at 4 °C. After electrophoresis, the bands of the biotin-labeled DNA were visualized using the ChemiDoc XRS+ (Bio-Rad, Hercules, CA, USA) molecular imager.

### 2.5. Determination of Cell Viability

*Spodoptera frugiperda* ovarian Sf9 cells were used to determine the cell viability and cultivated as described previously [21]. The BMB171 strain and Δ*nupR* strain were cultivated in Luria–Bertani (LB) medium to the T2 phase, after which the culture supernatant was collected by centrifugation. The supernatant was sterilized and diluted 10 times with PBS. Subsequently, 20 microliters of the diluted supernatant were added to 200 microliters of Sf9 insect cell culture. The cell viability was determined at 12-, 24-, and 48-h post-culture using the Cell Counting Kit-8 (CCK-8), following the manufacturer’s instructions.

### 2.6. Motility Assay

Overnight cultures were transferred to 100 mL of LB medium and cultured at 200 rpm and 28 °C until the cells reached the T2 phase. The samples at this point were used for motility assy. Swimming assays were performed on LB soft agar plates as described [22]. Freshly made soft agar (0.5% soft agar plates: 0.75 g agar/100 mL LB, 0.3% soft agar plates: 0.5 g agar/100 mL) was kept at 55 °C until the beginning of the assay. Twenty milliliters of soft agar were poured into a Petri dish and allowed to sterilize and dry for 10 min. Two microliters of the diluted culture were then spotted in the center of a Petri dish for inoculation, followed by incubation at 28 °C.

### 2.7. Statistical Analyses

The data were subjected to one-way analysis of variance using the Student’s *t*-test. Significance thresholds were specified as follows: * *p* < 0.05, ** *p* < 0.01, and *** *p* < 0.001, and ns, no significant. Data represent the mean ± SD of three samples.

## 3. Results

### 3.1. NupR Inhibits the Expression of plcR During the Stationary Phase

**Figure 1:**
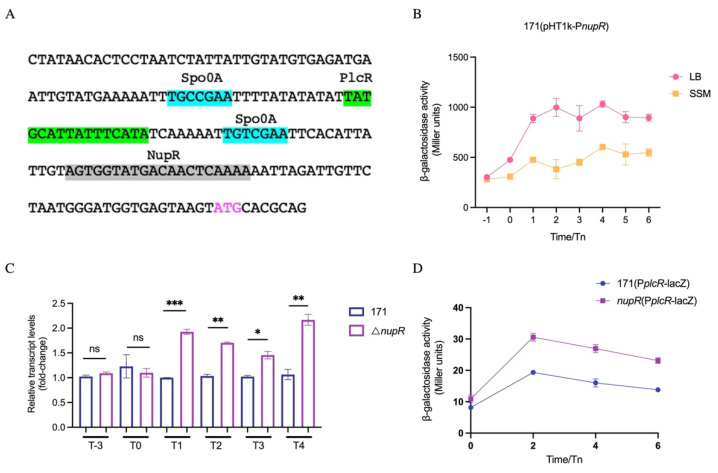
NupR inhibits the expression of *plcR* during the stationary phase. (**A**) The 5′UTR of *plcR*. The conserved binding sites of Spo0A, PlcR, and NupR are shaded in blue, green, and gray. The ATG of PlcR is marked in pink. (**B**) β-galactosidase activities of P*nupR*-*lacZ* in BMB171 cultivated in LB and SSM media. (**C**) *plcR* mRNA levels in BMB171 and Δ*nupR* cultivated in LB medium. (**D**) β-galactosidase activities of P*plcR*-*lacZ* in BMB171 and Δ*nupR* cultivated in LB medium. * *p* < 0.05; ** *p* < 0.01; *** *p* < 0.001; ns, non-significant. Data represent the mean ± SD of three samples.

Previous results showed that NupR directly binds to the intergenic region of *plcR* and *baci* [16]. Based on the NupR boxes, we predicted the DNA sequence (AGTGGTATGACAACTCAAAA) that NupR directly binds to, which is located upstream of the RBS of *plcR* (Figure 1A), closer to the start codon of *plcR* than that of *baci*. Therefore, we speculate that NupR directly regulates the expression of *plcR*. To verify this hypothesis, we first determined the expression phase of *nupR*. The results showed that in either rich medium LB or minimal medium SSM, the promoter activity of *nupR* reached the highest level at T1, suggesting that it mainly plays a regulatory role during the stationary phase (Figure 1B).

To explore the difference in *plcR* expression between the BMB171 strain and the Δ*nupR* strain, we measured the mRNA levels of *plcR* in both strains. The results showed that the mRNA level of *plcR* in the Δ*nupR* strain was significantly higher than that in the BMB171 strain during the stationary phase (Figure 1C). In addition, we also connected the promoter and 5’ noncoding region of *plcR* to the *lacZ* reporter gene and transferred it into the BMB171 strain and the Δ*nupR* strain. The β-galactosidase activity assay results showed that the activity of the *plcR* promoter in the BMB171 strain was significantly lower than that in the Δ*nupR* strain, which is consistent with the qRT-PCR results (Figure 1D). In summary, the expression of *plcR* is negatively regulated by NupR during the stationary phase.

### 3.2. NupR Can Directly or Indirectly Regulate the Expression of the plcR Regulon

**Figure 2:**
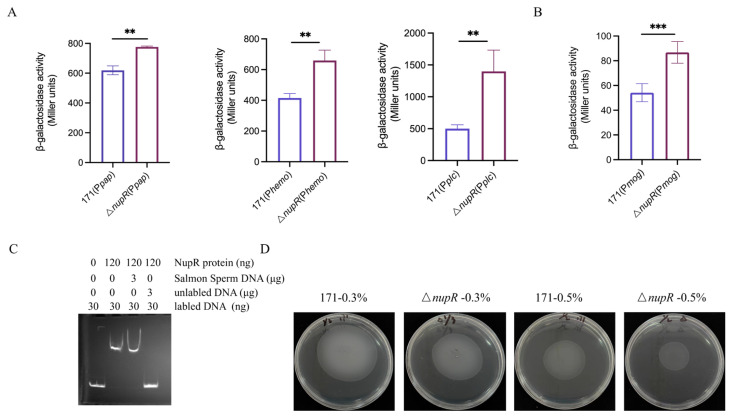
NupR can directly or indirectly regulate the expression of the *plcR* regulon. (**A**) β-galactosidase activities of P*pap*-lacZ, P*hemo*-lacZ, and P*plc*-lacZ in BMB171 and Δ*nupR* cultivated in the SSM medium. (**B**) β-galactosidase activities of P*mog*-lacZ in BMB171 and Δ*nupR* cultivated in the SSM medium. (**C**) NupR binds directly to the promoter regions of *mogR* labeled by FAM. (**D**) BMB171, and Δ*nupR* strains were dripped on 0.5% and 0.3% soft agar plates (LB medium) and incubated at 28 °C. ** *p* < 0.01; *** *p* < 0.001. Data represent the mean ± SD of three samples.

Given that NupR directly downregulates the expression of *plcR*, it may indirectly control the transcription of PlcR-dependent genes. It has been reported that PlcR positively regulates the transcription of various virulence genes, such as *phospholipase C* [23], *hemolysin* [4], and *papR* [2]. Therefore, expression vectors with the promoters of these genes fused to *lacZ* were constructed and introduced into the BMB171 strain and the Δ*nupR* strain to measure β-galactosidase activity. The results showed that the *plc*, *hemolysin*, and *papR* promoter activities significantly increased in the Δ*nupR* strain (Figure 2A). NupR may reduce the transcription of these three genes by inhibiting *plcR*.

*mogR* is the only gene experimentally verified to be directly downregulated by PlcR, which encodes the motility gene repressor protein [24,25]. The activity of the *mogR* promoter was measured in the BMB171 strain and the Δ*nupR* strain. The results indicated that the promoter activity was significantly increased in the Δ*nupR* strain, suggesting that NupR represses *mogR* expression (Figure 2B).

If NupR modulates the expression of *mogR* through PlcR, the expression of *mogR* in the Δ*nupR* strain should theoretically be downregulated. However, the experimental results failed to confirm this hypothesis. Thus, an EMSA assay was conducted to investigate the regulation of *mogR* gene expression by NupR. The result indicated that the NupR protein can directly bind to the promoter sequence of the *mogR* gene (Figure 2C). Thus, NupR can directly inhibit the expression of *mogR*. Additionally, the impact of the *nupR* deletion on the motility of the strain was measured. The results showed that the motility of the Δ*nupR* was significantly reduced (Figure 2D). MogR represses the synthesis of flagella, so the reduction in motility may be due to the upregulation of *mogR* expression in Δ*nupR*, which is consistent with the enzymatic activity results.

### 3.3. Effect of nupR Deletion on the Virulence of Bacillus thuringiensis

**Figure 3:**
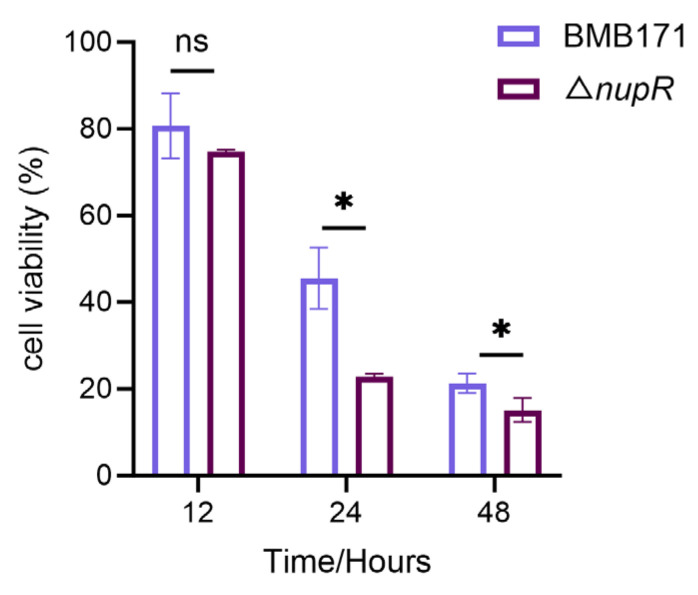
The effect of the culture supernatant of BMB171 and Δ*nupR* strains on the cell viability of Sf9 cells. * *p* < 0.05; ns, no significant. Data represent the mean ± SD of three samples.

Since NupR influences the expression of virulence factors, the deletion of *nupR* may affect the production of virulence factors of the strain, which can change the virulence of the strain. The *Bacillus thuringiensis* in this study did not contain insecticidal proteins. The toxicity of the strain culture supernatant in the stable phase was determined against *S. frugiperda* Sf9 cells, the ovary cells of the grass-coveting nightshade moth, to test the effect of *nupR* deletion on the virulence of the BMB171 strain. The results showed that the toxicity of the Δ*nupR* supernatant was higher than that of the BMB171 strain at 24 h and 48 h (Figure 3). Therefore, NupR may attenuate its virulence by inhibiting the expression of the cytotoxicity regulator.

### 3.4. Expression of plcR Is Induced by Glucose

**Figure 4:**
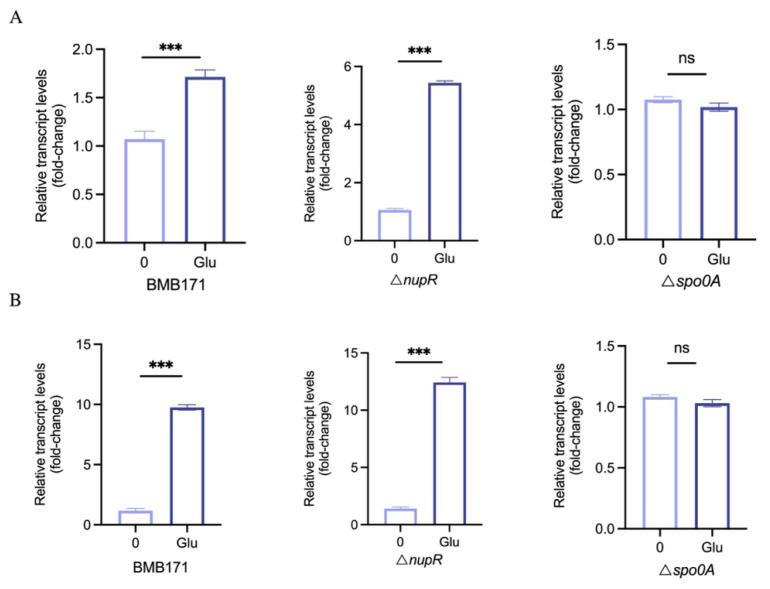
Expression of *plcR* is induced by glucose. (**A**) *plcR* mRNA levels in BMB171, Δ*nupR*, and Δ*spo0A* cultivated in SSM medium with or without 0.1% glucose. (**B**) *plcR* mRNA levels in BMB171, Δ*nupR*, and Δ*spo0A* cultivated in LB medium with or without 0.1% glucose. *** *p* < 0.001; ns, no significant. Data represent the mean ± SD of three samples.

The expression of *nupR* is positively regulated by CcpA and is induced significantly by glucose [16]. NupR directly inhibits the expression of *plcR*, suggesting that the expression of *plcR* may be repressed by glucose. The effect of glucose on *plcR* expression was determined. The strains were cultured in LB or SSM medium until the early stationary phase and induced with 0.1% glucose for 30 min. The mRNA levels of *plcR* in the BMB171 strain and the Δ*nupR* strain were measured. The results showed that in the SSM medium, the mRNA level of *plcR* was upregulated 1.7-fold in the BMB171 strain and 5.3-fold in the Δ*nupR* strain. In the LB medium, it was upregulated 9.5-fold in the BMB171 strain and 12.5-fold in the Δ*nupR* strain (Figure 4). After the deletion of *nupR*, the expression of *plcR* was no longer inhibited by NupR. It became more sensitive to glucose, with a higher fold increase in expression than the BMB171 strain.

**Figure 5:**
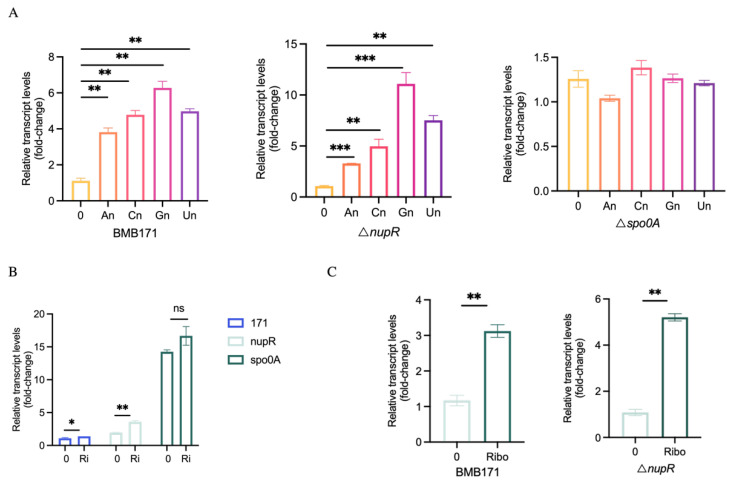
Expression of *plcR* is Induced by Nucleosides. (**A**) *plcR* mRNA levels in BMB171, Δ*nupR*, and Δ*spo0A* cultivated in SSM medium with or without 1mM different nucleosides. An, adenosine; Cn, cytidine; Gn, guanosine; Un, uridine. (**B**) *plcR* mRNA levels in BMB171, Δ*nupR*, and Δ*spo0A* cultivated in SSM medium with or without 0.1% ribose. (**C**) *plcR* mRNA levels in BMB171 and Δ*nupR* cultivated in LB medium with or without 0.1% ribose. Ri, ribose. * *p* < 0.05; ** *p* < 0.01; *** *p* < 0.001; ns, no significant. Data represent the mean ± SD of three samples.

Glucose still induces the expression of *plcR* in the Δ*nupR* strain. Therefore, the promoting effect of glucose on the expression of *plcR* is evidently due to other reasons. It has been reported that the expression of *plcR* is inhibited by Spo0A. A conserved binding site for Spo0A was predicted in the promoter region of *plcR* (Figure 1A). Moreover, the mRNA level of *plcR* in the Δ*spo0A* strain is 15 times higher than in the BMB171 strain (Figure 5B). The additional glucose may promote the expression of *plcR* by reducing the inhibition of Spo0A. The mRNA level of *plcR* in the Δ*spo0A* strain under glucose induction was measured. The results showed that in the absence of *spo0A*, the expression of *plcR* was no longer promoted by glucose (Figure 4). However, the expression level of *spo0A* in the BMB171 strain did not change in the presence of glucose, leading us to speculate that glucose may reduce the phosphorylation of Spo0A, weakening its inhibition of *plcR* and resulting in an increase in the expression of *plcR*.

### 3.5. Expression of plcR Is Induced by Nucleosides

*nupR* encodes a regulator of nucleoside permeases, affecting the utilization of guanosine, adenosine, uridine, and cytidine [16]. Therefore, we hypothesized that nucleosides may act as regulators in virulence modulation. Like the induction method with glucose, after adding 1 mM of different nucleosides to the culture medium, the mRNA levels of *plcR* in both the BMB171 strain and the Δ*nupR* strain were measured. The results showed that the expression of *plcR* in both strains significantly increased after adding different nucleosides, with guanosine showing the most pronounced effect (Figure 5A). The expression of *plcR* was upregulated 6-fold in the BMB171 strain and 10-fold in the Δ*nupR* strain by guanosine. Since all these nucleosides contain ribose, we speculate that the effect of nucleosides on *plcR* may primarily be due to ribose.

Similarly, after adding 0.1% ribose to the culture medium, the expression level of *plcR* was measured. The results showed that the expression of *plcR* increased in both strains after adding ribose. In the SSM medium, the mRNA level of *plcR* was upregulated 1.3-fold in the BMB171 strain and 1.9-fold in the Δ*nupR* strain (Figure 5B). In the LB medium, the mRNA level of *plcR* was upregulated three-fold in the BMB171 strain and five-fold in the Δ*nupR* strain (Figure 5C). In addition, the effect of ribose on *plcR* expression was measured in the Δ*spo0A* strain, and the results showed that after the deletion of *spo0A*, ribose no longer affected the expression of *plcR* (Figure 5B). Overall, Spo0A plays a leading role in glucose and ribose induction.

## 4. Discussion

This study discovered that the nucleoside permease regulator NupR can directly inhibit the transcription of *plcR* during the stationary phase, thereby impacting the expression of PlcR-dependent virulence factors. After *nupR* deletion, the strain supernatant’s toxicity to Sf9 cells was significantly enhanced. This further proves the regulatory role of NupR on the virulence of Bt.

Six genes encoding oligopeptide permease were found to be differentially expressed in the transcriptomic data of Δ*nupR* and BMB171. Oligopeptide permeases are ATP-binding cassette transporters consisting of five proteins: two membrane-integrating proteins that form the actual pore (OppB and OppC), two ATPases that bind to the membrane proteins to provide the energy required for transport (OppD and OppF), and an extracellularly oriented, membrane-anchored substrate-binding protein (SBP) (OppA). OppBCDF has been reported to be required for PapR import [26,27]. In addition, OppA is not the only SBP involved in recognizing PapR, and several other OppA-like proteins can import this peptide [28]. Therefore, oligopeptide permease is indispensable in PlcR activation. The mRNA levels of these six permeases were determined in the BMB171 strain and the Δ*nupR* strain. The results showed that two permease (*oppAs*) genes were significantly upregulated during the stable phase (Figure S1). Therefore, after *nupR* deletion, the mRNA level of *plcR* was elevated, and its activation efficiency was also likely increased by the high expression of the oligopeptide permeases and *papR*.

The expression of *plcR* is induced by glucose, and its induction fold increased after *nupR* was deleted. After the deletion of *spo0A*, *plcR* expression was no longer affected. Therefore, the inducing effect of *plcR* by glucose is caused by Spo0A. NupR does, indeed, have an inhibitory effect on the expression of *plcR* under the action of glucose, but this effect is no longer evident after the deletion of *spo0A*. In the absence of *spo0A*, the expression of *plcR* is upregulated approximately 15 times. In this case, the inhibitory effect of NupR becomes insignificant. As a regulator of nucleoside permeases, NupR can directly affect the utilization of nucleosides by bacteria. The expression of *plcR* is significantly increased after adding different nucleosides, and the breakdown product of nucleosides, ribose, may cause this effect.

The global regulatory factor CodY, prevalent in low-GC-content gram-positive bacteria, regulates early stationary phase genes and initiates sporulation [29]. CodY positively regulates the expression of oligopeptide permeases, oppABCDF, and several other Opp-like proteins, which influence the transport of PapR, leading to the activation of PlcR [28]. Consequently, CodY exerts a stimulatory effect on the transcription of PlcR-dependent genes. In contrast to CodY, NupR exerts a suppressive influence on the expression of *oppA*, inhibiting the activation of *plcR*. Hence, the regulatory impacts of CodY and NupR on *plcR* expression are antagonistic. When the culture medium is replete with glucose, resulting in reduced levels of branched-chain amino acids (BCAAs), the function of CodY may be diminished [29]. Consequently, in the presence of glucose, the expression of the *plcR* gene may be reduced via CodY.

**Figure 6:**
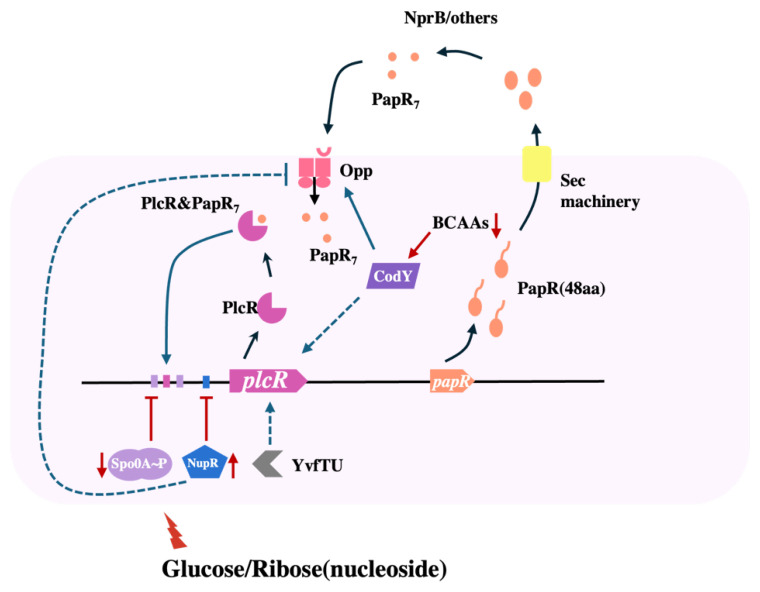
Schematic representation of the regulation of PlcR. *plcR* is autoregulated and under the negative control of Spo0A~P and NupR. CodY positively controls the expression of *plcR* by regulating the expression of *opp*. The YvfTU two-component system is also involved in *plcR* expression via a yet unknown mechanism. Process lines are shown in black, and regulatory relationships are indicated by blue and red lines, with clipped heads for facilitation, horizontal lines for inhibition, solid lines for direct, and dashed lines for indirect. In addition, the red lines indicate processes influenced by ribose or glucose.

The pleiotropic regulator PlcR promotes the transcription of virulence factor genes during the stationary phase, such as degradation enzymes, antibiotics, toxins, etc., ensuring the production of specific compounds necessary for spore formation [9]. Figure 6 shows a schematic representation of the regulation of PlcR based on diagrams drawn by Slamti et al. [30]. The expression of *plcR* is controlled by Spo0A and the nucleoside permease regulator NupR. As bacteria enter the late exponential phase and the nutrients in the culture medium are consumed, the strain transitions from exponential growth to a transitional state, where the concentration of Spo0A-P increases sharply and represses *plcR* expression [31,32,33]. NupR further suppresses the expression of *plcR* during the stationary phase. If additional glucose is added, it could reduce the concentration of Spo0A-P by inhibiting the Calvin cycle, resulting in a high expression of *plcR* [34]. Glucose will also suppress the expression of *plcR* by inducing the expression of NupR, compensating for the reduced effect of Spo0A. Besides, the promotion of *plcR* by CodY is diminished under glucose conditions. NupR and CodY are auxiliary modulators within this regulatory framework, whereas Spo0A assumes the primary regulatory function. This is underscored by the observation that following the deletion of *spo0A*, the expression of *plcR* becomes independent of glucose influence.

This research identifies the regulator NupR as a component of the regulatory network governing *plcR*, which directly modulates *plcR* expression and may also exert indirect effects on PlcR activation through the regulation of *oppA* expression. It has significant implications for the virulence of the BMB171 strain. We elucidated, for the first time, the role of NupR in regulating virulence. Furthermore, we demonstrated that *plcR* expression can be induced by glucose and nucleosides, highlighting the nuanced regulatory role of NupR and the decisive role of Spo0A in this process. This regulatory circuit may establish a connection between strain virulence, nutritional conditions, and fate decisions, thereby coordinating bacterial behavior. Besides, this study revealed that the additional inclusion of glucose and nucleosides in the LB medium or the *nupR* deletion significantly enhances the expression of *plcR*, thereby increasing the virulence of the bacteria. This finding provides a theoretical foundation for future studies on bacterial toxin production or related research.

## 5. Conclusions

We determined that *plcR* is regulated by NupR (nucleoside permease regulator), a member of the GntR family, which is a novel component of the regulatory network governing *plcR*. Besides, additional glucose and nucleosides can induce *plcR* expression mainly through Spo0A. This regulatory circuit may establish a connection between strain virulence, nutritional conditions, and fate decisions, thereby coordinating bacterial behavior.

## Data Availability

The data supporting the findings of this study are available at Figshare (https://doi.org/10.6084/m9.figshare.27211629).

## Supplementary Materials

The following supporting information can be downloaded online: https://www.mdpi.com/article/10.3390/microorganisms13010212/s1. Figure S1: *opp* mRNA levels in BMB171 and Δ*nupR* cultivated in SSM medium (T2); Table S1: Bacterial strains and plasmids used in this study. References [16,35] are cited in the supplementary materials.
