# Supplementary material for: NupR Is Involved in the Control of PlcR: A Pleiotropic Regulator of Extracellular Virulence Factors
Source: Microorganisms. 2025 Jan 20;13(1):212. doi: 10.3390/microorganisms13010212 (PMC11767619; doi:10.3390/microorganisms13010212)
Supplement: Supplementary file 1 [file microorganisms-13-00212-s001.zip › microorganisms-3379179-supplementary.pdf]

Figure S1

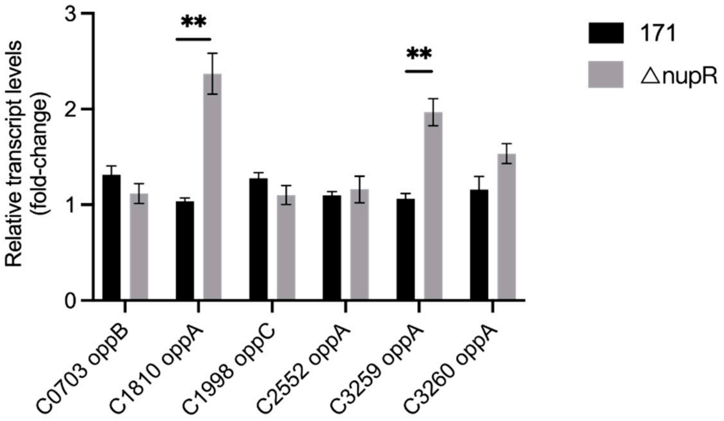

Figure S1. *opp* mRNA levels in BMB171 and △*nupR* cultivated in SSM medium (T2). **P < 0.01.

Table S1 Bacterial strains, plasmids used in this study.

Strain/ Plasmid	Description	purposes	Source (Reference）
**Stains**			
BMB171	B. thuringiensis strain BMB171; an acrystalliferous mutant strain; high transformation frequency		(He et al., 2010)
△*nupR*	BMB171 deleted of *nupR*		(Qin et al., 2022)
BMB171(pRB1028 -*spo0A*)	BMB171 containing plasmid pRB1028-*spo0A*	gene-knockout	this study[1]
△*spo0A*	BMB171 deleted of *spo0A*		this study
BMB171(pHT1K-P*nupR*)	BMB171 containing plasmid pHT1K-P*nupR*	β-galactosidase assays	this study
BMB171(P*plcR*-lacZ)	BMB171 containing plasmid P*plcR*-lacZ	β-galactosidase assays	this study
△*nupR* (P*plcR*-lacZ)	△*nupR* containing plasmid P*plcR*-lacZ	β-galactosidase assays	this study
171(P*pap*)	BMB171 containing plasmid P*pap*	β-galactosidase assays	this study
△*nupR* (P*pap*)	△*nupR* containing plasmid P*pap*	β-galactosidase assays	this study
171(P*hemo*)	BMB171 containing plasmid P*hemo*	β-galactosidase assays	this study
△*nupR* (P*hemo*)	△*nupR* containing plasmid P*hemo*	β-galactosidase assays	this study
171(P*plc*)	BMB171 containing plasmid P*plc*	β-galactosidase assays	this study
△*nupR* (P*plc*)	△*nupR* containing plasmid P*plc*	β-galactosidase assays	this study
171(P*mog*)	BMB171 containing plasmid P*mog*	β-galactosidase	this study

		assays	
△*nupR* (*mog*)	△*nupR* containing plasmid P*mog*	β-galactosidase assays	this study
BL21(pET*nupR*)	BL21(DE3) with pET*nagR2* plasmid	protain purification	(Qin et al., 2022)
**Plasmid**			
pHT1K-lacZ	B. thuringiensis-*E. coli* shuttle plasmid; Amp^R Erm^R, pHT1K vector harboring the promoter-less *lacZ* gene, transformed into BMB171 and used for β-galactosidase activity	β-galactosidase assays	
P*plcR*-lacZ	lacZ with the promoter and the 5' non-coding region of *plcR* in Nco I and Bam HI sites of pHT1K	β-galactosidase assays	this study
P*pap*	lacZ with the promoter and the 5' non-coding region of *pap* in Nco I and Bam HI sites of pHT1K	β-galactosidase assays	this study
P*hemo*	lacZ with the promoter and the 5' non-coding region of *hemo* in Nco I and Bam HI sites of pHT1K	β-galactosidase assays	this study
P*plc*	lacZ with the promoter and the 5' non-coding region of *plc* in Nco I and Bam HI sites of pHT1K	β-galactosidase assays	this study
P*mog*	lacZ with the promoter and the 5' non-coding region of *mogR* in Nco I and Bam HI sites of pHT1K	β-galactosidase assays	this study
pRP1028	*B. thuringiensis-E. coli* shuttle plasmid; Amp^R Erm^R; containing *turbo-rfp* gene and an I-*Sce* I recognition site	gene-knockout	
pRP1028-*spo0A*	pRP1028 with the upstream and downstream regions of *spo0A*, intermediate vector in gene-knockout experiments	gene-knockout	this study

[1] the stain or plasmid is constructed for this research and stored in our laboratory at -80°C

He, J., Shao, X., Zheng, H., Li, M., Wang, J., Zhang, Q., Li, L., Liu, Z., Sun, M., Wang, S., Yu, Z. Complete genome sequence of Bacillus thuringiensis mutant strain BMB171. J Bacteriol. 2010 Aug;192(15):4074-5.

Qin, J., Cao, Z., Cai, X., Fang, Y., An, B., Li, X., Zhang, Y., Tian, H., Hu, W., Yan, B., & Cai, J. (2022). NupR Responding to Multiple Signals Is a Nucleoside Permease Regulator in Bacillus thuringiensis BMB171. Microbiology spectrum, 10(4), e0154322.
